# A systematic review of the comparison of the incidence of seeding metastasis between endoscopic biliary drainage and percutaneous transhepatic biliary drainage for resectable malignant biliary obstruction

**DOI:** 10.1186/s12957-019-1656-y

**Published:** 2019-07-05

**Authors:** Lei Wang, Nanping Lin, Fuli Xin, Qiao Ke, Yongyi Zeng, Jingfeng Liu

**Affiliations:** 1 The United Innovation of Mengchao Hepatobiliary Technology Key Laboratory of Fujian Province, Mengchao Hepatobiliary Hospital of Fujian Medical University, Fuzhou, 350025 Fujian People’s Republic of China; 2 The Liver Center of Fujian Province, Fujian Medical University, Fuzhou, 350025 Fujian People’s Republic of China; 3 The First Clinical Medical College of Fujian Medical University, Fuzhou, 350005 Fujian People’s Republic of China; 4 Liver Disease Center, The First Affiliated Hospital of Fujian Medical University, Fuzhou, 350007 Fujian People’s Republic of China; 5 Mengchao Hepatobiliary Hospital of Fujian Medical University, Xihong Road 312, Fuzhou, 350025 Fujian People’s Republic of China

**Keywords:** Preoperative biliary drainage, Malignant biliary obstruction, Percutaneous biliary drainage, Endoscope biliary drainage, Seeding metastasis, Meta-analysis

## Abstract

**Background and aim:**

Endoscopic biliary drainage (EBD) and percutaneous biliary drainage (PTBD) are the two main strategies of preoperative biliary drainage (PBD) for resectable malignant biliary obstruction (MBO) worldwide, but which is better remains unclear. Seeding metastasis (SM) has been reported repeatedly in the recent decade, although it is rarely taken into consideration in the choice of PBD. Hence, a systematic review was badly warranted to evaluate the incidence of SM between PTBD and EBD in the preoperative treatment of MBO.

**Methods:**

PubMed, MEDLINE, the Cochrane Library, and Web of Science were used to identify any potentially eligible studies comparing the incidence of SM between EBD and PTBD from Nov 1990 to Mar 2018. The effect size was determined by odds ratio (OR) with 95% confidence interval (CI).

**Results:**

Ten studies were enrolled in this study, including 1379 cases in the EBD group and 1085 cases in the PTBD group. Results showed that the incidence of SM in the EBD group was significantly lower than that in the PTBD group (10.5% vs. 22.0%, OR = 0.35, 95% CI 0.23~0.53). Subgroup analysis stratified by the definition of SM showed that the pooled ORs for peritoneal metastasis and tube-related SM between EBD and PTBD were 0.42 (95% CI 0.31~0.57) and 0.17 (95% CI 0.10~0.29), respectively. Subgroup analysis stratified by the location of MBO showed that the pooled ORs for the incidence of SM between EBD and PTBD for perihilar cholangiocarcinoma, distal cholangiocarcinoma, and pancreatic cancer were 0.27 (95% CI 0.13~0.56), 0.32 (95% CI 0.17~0.60), and 0.27 (95% CI 0.19~0.40), respectively.

**Conclusion:**

EBD should be the optimal PBD for MBO considering the SM, but it deserved further validation.

## Introduction

Patients diagnosed with perihilar cholangiocarcinoma (PHC), distal cholangiocarcinoma (DCC), and pancreatic cancer (PC) typically present with malignant biliary obstruction (MBO), which is one of the crucial reasons for the failure of surgery [1]. Preoperative biliary drainage (PBD) is deemed to improve jaundice before surgery and decrease postoperative morbidity and mortality, although it remains controversial [2–4]. Furthermore, either percutaneous transhepatic biliary drainage (PTBD) or endoscopic biliary drainage (EBD) is the best strategy for resectable MBO is also a question [5–9].

Seeding metastasis (SM) is rarely refereed worldwide, but it has been reported frequently in Japan [10–17]. The incidence of SM in Japan was reported to range from 4.0 to 40.4% [10–17], which is no longer an “unusual” contingency. EBD was reported repeatedly to superior to PTBD in the prophylaxis of SM [10–17], but it was contradicted by a multicenter, retrospective study derived from US-Euro [18]. Hence, a systematic review is warranted to evaluate the incidence of SM between EBD and PTBD in the procedure of PBD for patients with MBO.

## Materials and methods

### Literature search

A comprehensive search was conducted by two independent researchers to clarify all published researches of PBD for preoperative obstructive jaundice. English electronic databases such as PubMed, MEDLINE, the Cochrane Library, and Web of Science were used to seek the literature, from Nov 1990 to Mar 2018. Keywords including “preoperative biliary drainage” and “malignant biliary obstruction” combined with free text words such as “percutaneous transhepatic biliary drainage” or “endoscopic biliary drainage” or “seeding metastasis” appeared in the electronic search.

### Selection criteria

Inclusion criteria are as follows: (1) cohort studies and randomized controlled trials were both considered, (2) PBD either PTBD or EBD for patients with MBO, (3) the primary endpoint was SM, and (4) sufficient data such as the baseline of characteristic were depicted.

Exclusion criteria are as follows: (1) in vitro or animal studies; (2) case reports, letters, reviews, and conference reports; (3) studies based on overlapping cohorts derived from the same center; and (4) sample size was not more than 20.

In case of results reported from the same center more than once, the latest was extracted.

### Data extraction

Predefined forms including baseline characteristics and outcomes were extracted from each study by Nanping Lin and Fuli Xin independently and then assessed by Lei Wang, Nanping Lin, and Fuli Xin. In the case of disagreement, a third investigator intervened for a conclusion.

### Intervention and outcome definition

PTBD (percutaneous transhepatic biliary drainage), including external drainage and internal drainage (percutaneous transhepatic biliary stent, PTBS), is depicted in Table 2.

EBD (endoscopic biliary drainage), including external drainage such as endoscopic nasobiliary drainage (ENBD) and internal drainage (endoscopic biliary stent, EBS), is also depicted in Table 2.

The mean interval between surgery and onset of the recurrence (SM) is depicted in Table 1.

**Table 1:** Clinicopathological characteristics of trials included

Study	Country	Study year	Design of studies	Follow-up(months)	Tumor type	PTBD				EBD				Outcome indicators
						No.	TBIL (mg/dl)	Histologic grade	Tumor location	No.	TBIL (mg/dl)	Histologic grade	Tumor location	
								Poorly differentiated				Poorly differentiated		
Kawakami et al. [10]	Japan	1999–2009	RCS	60	PHC	48	12.0	–	I 4 II 12 IIIa 8 IIIb 8 IV 16	80	9.6	–	I 15 II 22 IIIa 16 IIIb 11 IV 16	①, ②
Hwang et al. [19]	Korea	1985–2002	RCS	120	PHC	171	–	–	–	62	–	–	–	①, ②
Murakami et al. [11]	Japan	1998–2013	RCS	60	PC	20	–	14	–	73	–	47	–	①, ②, ③
Hirano et al. [12]	Japan	2000–2008	RCS	160	PHC	67	8.4	–	I 5 II 16 IIIa 13 IIIb 13 IV 20	74	5.2	–	I 15 II 21 IIIa 12 IIIb 16 IV 10	①, ②, ③
Komaya et al. [13]	Japan	2001–2010	RCS	60	DCC	189	7.4	117	Middle 53 Low 136	187	4.7	123	Middle 61 Low 126	①, ②, ③
Uemura et al. [14]	Japan	2001–2012	RCS	120	PC	166	–	163	–	407	–	392	–	①, ②, ③, ④
Wiggers et al. [18]	Netherland/USA	1991–2012	RCS	60	PC	88	11.2	–	I 8 II 11 IIIa 30 IIIb 18 IV:19	157	3.2	–	I 41 II 23 IIIa 44 IIIb 28 IV 16	①, ③
Komaya et al. [15]	Japan	2003–2012	RCS	60	PHC	168	–	123	I/II/III 77 IV 91	152	–	113	I/II/III 92 IV 60	①, ②, ③
Higuchi et al. [16]	Japan	2000–2013	RCS	12~60	PHC	87	–	58	I/II/III 50 IV 37	76	–	52	I/II/III 44 IV 32	①, ②
Miura et al. [17]	Japan	1987–2015	RCS	60	DCC	25	3.7	12	–	63	2.4	32	–	①, ②

Bismuth’s classification: subtypes I, II, III, and IV; outcome indicators: ① PTBD catheter tract recurrence, ② pleural dissemination on the right side alone, ③ peritoneal dissemination, and ④ intrahepatic metastasis (only for PC)

*TBIL* total bilirubin, *BC* Bismuth classification, *PHC* perihilar cholangiocarcinoma, *DCC* distal cholangiocarcinoma, *PC* pancreatic head carcinoma, *RCS* retrospective cohort studies, *NOS* Newcastle-Ottawa Scale, “–” not mentioned

SM was extracted directly from the original studies and was different from each other. The types of SM were as follows: (1) PTBD catheter tract recurrence, (2) pleural dissemination on the right side alone, (3) peritoneal dissemination, and (4) intrahepatic metastasis (only for PC) [10, 14].

When it was hard to distinguish tube-related SM with peritoneal metastasis, data was merged and subgroup analysis was avoided.

### Quality assessment

Considering all of the studies were retrospective cohort studies, quality assessment was assessed by the Newcastle-Ottawa Scale (NOS). Studies scored as ≥ 6 were considered of high quality.

### Statistical analysis

The systematic review was registered at http://www.crd.york.ac.uk (122086) and performed using RevMan version 5.3 and Stata 14. Considering the apparent heterogeneity among different studies, such as different strategies of PBD, the stent material of biliary drainage, and the severity of obstructive jaundice, the random-effects model was used to compare the incidence of seeding metastasis between PTBD and EBD [20]. Odds ratios (ORs) were for the dichotomous outcomes, followed with 95% confidence intervals (CI). Publication bias was evaluated by visually assessing the asymmetry of an inverted funnel plot and then was supported quantitatively by Egger’s tests.

## Results

### Base characteristic of the included studies

**Fig. 1:**
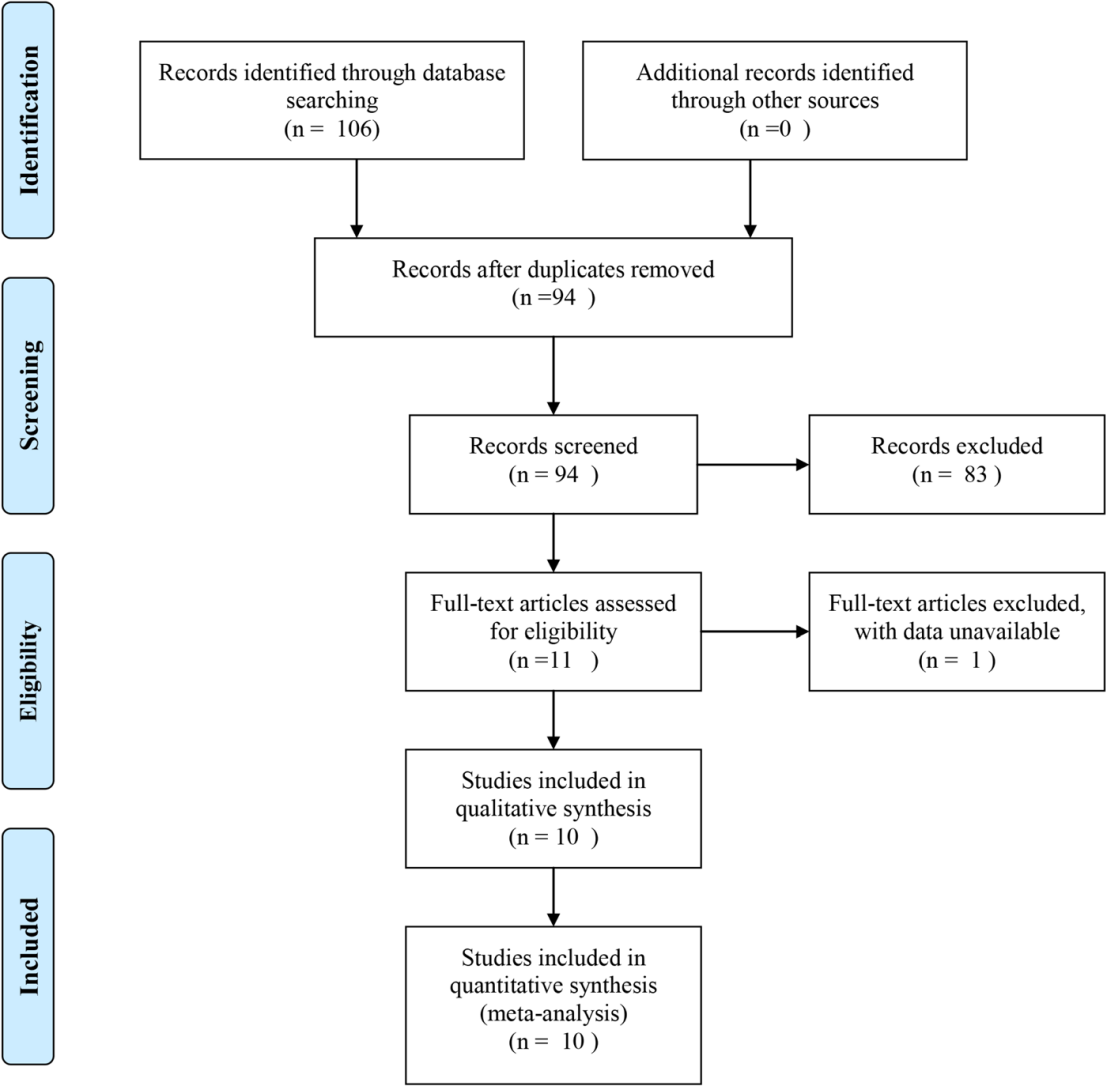
Flowchart of the study selection process for meta-analysis

Initially, 106 reports were identified by two independent reviewers. Twelve articles were excluded after duplicate removal by NoteExpress 3.1. After browsing titles and abstracts, 83 records were excluded. Among the remaining 11 articles, one record was excluded for lack of enough cases. Finally, 10 reports remained, including 6 studies of PHC [10, 12, 15, 16, 18, 19], 2 of DCC, and 2 of PC [11, 14]. In total, 2464 patients were enrolled in this meta-analysis, with 1379 cases in the EBD group and 1085 cases in the PTBD group (Fig. 1).

The characteristic and quality of the included trials are shown in Table 1. All the studies included in this meta-analysis were nonrandomized studies and were assessed by NOS. The scores ranged from 6 to 9, which indicated that all the studies were of high quality (Table 2).

**Table 2:** Newcastle-Ottawa quality assessment of the included studies

Study	Selection				Comparability	Outcome			Scores
	Representativeness of the exposed cohort	Selection of the non-exposed cohort	Ascertainment of exposure	Outcome of interest was presented		Assessment of outcome	Follow-up long enough for outcomes to occur	Adequacy of follow-up of cohorts	
Kawakami et al. [10]	★	★	★	★	★	★	★	★	8
Hwang et al. [19]	★	★	★	★	★	★	★		7
Murakami et al. [11]	★	★	★		★	★	★	★	7
Hirano et al. [12]	★	★	★	★	★		★	★	7
Komaya et al. [13]	★	★	★		★★	★	★	★	8
Uemura et al. [14]	★	★	★		★★	★	★	★	8
Wiggers et al. [18]	★		★	★		★	★	★	6
Komaya et al. [15]	★	★	★	★	★★	★	★	★	9
Higuchi et al. [16]	★	★	★	★	★★	★	★	★	9
Miura et al. [17]	★	★	★		★	★	★	★	7

^★^Score of the paper got after assessment

### Comparison of SM incidence between EBD and PTBD for resectable MBO

**Fig. 2:**
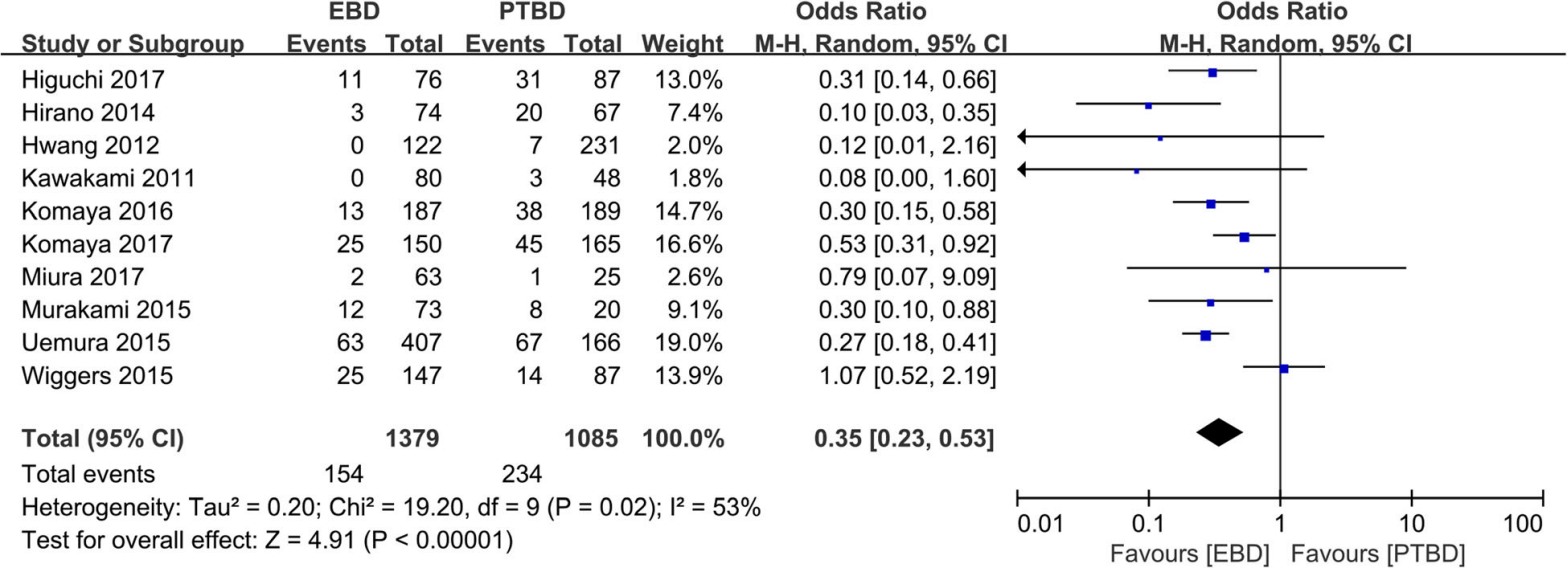
Forest plots of the seeding metastasis rates

SM was reported in all the included studies [10–19], and results showed that there were significant differences in the rates of seeding metastasis between EBD and PTBD (10.5% vs. 22.0%, OR = 0.35, 95% CI 0.23~0.53, *P* < 0.001, Fig. 2).

### Subgroup analysis of different SM incidences between EBD and PTBD for resectable MBO

**Fig. 3:**
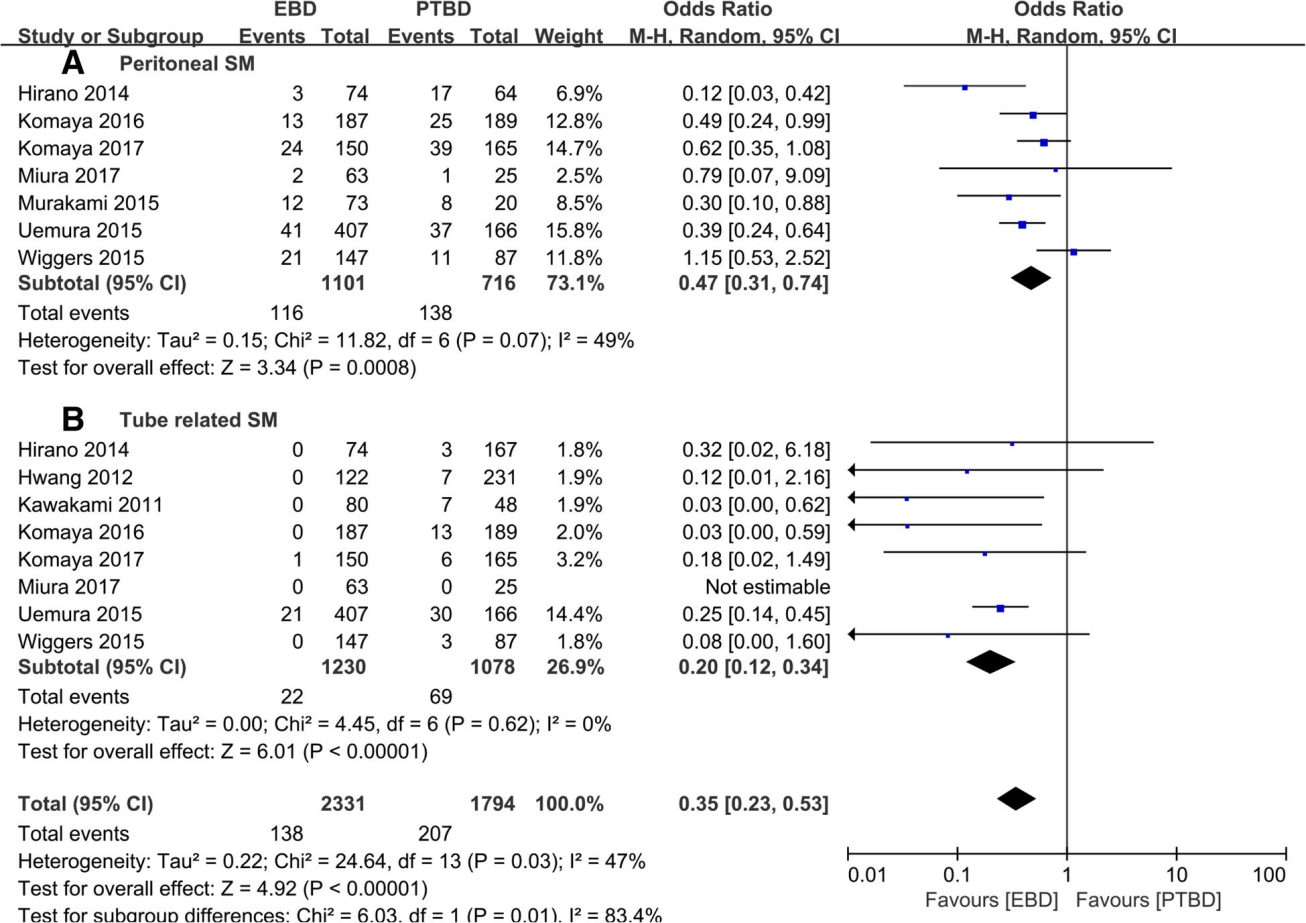
Subgroup analysis of (**a**) peritoneal metastasis and (**b**) tube-related seeding metastasis

SM was divided into peritoneal metastasis and tube-related seeding metastasis, and subgroup results showed that EBD was superior to PTBD both in peritoneal metastasis [11–15, 17, 18] (10.0% vs. 20.2%, OR = 0.42, 95% CI 0.31~0.57, *P* < 0.001, Fig. 3(a)) and tube-related SM [10, 12–15, 17–19] (2.0% vs. 6.7%, OR = 0.17, 95% CI 0.10~0.29, *P* < 0.001, Fig. 3(b)).

### Subgroup analysis of SM incidences between EBD and PTBD for different MBO

**Fig. 4:**
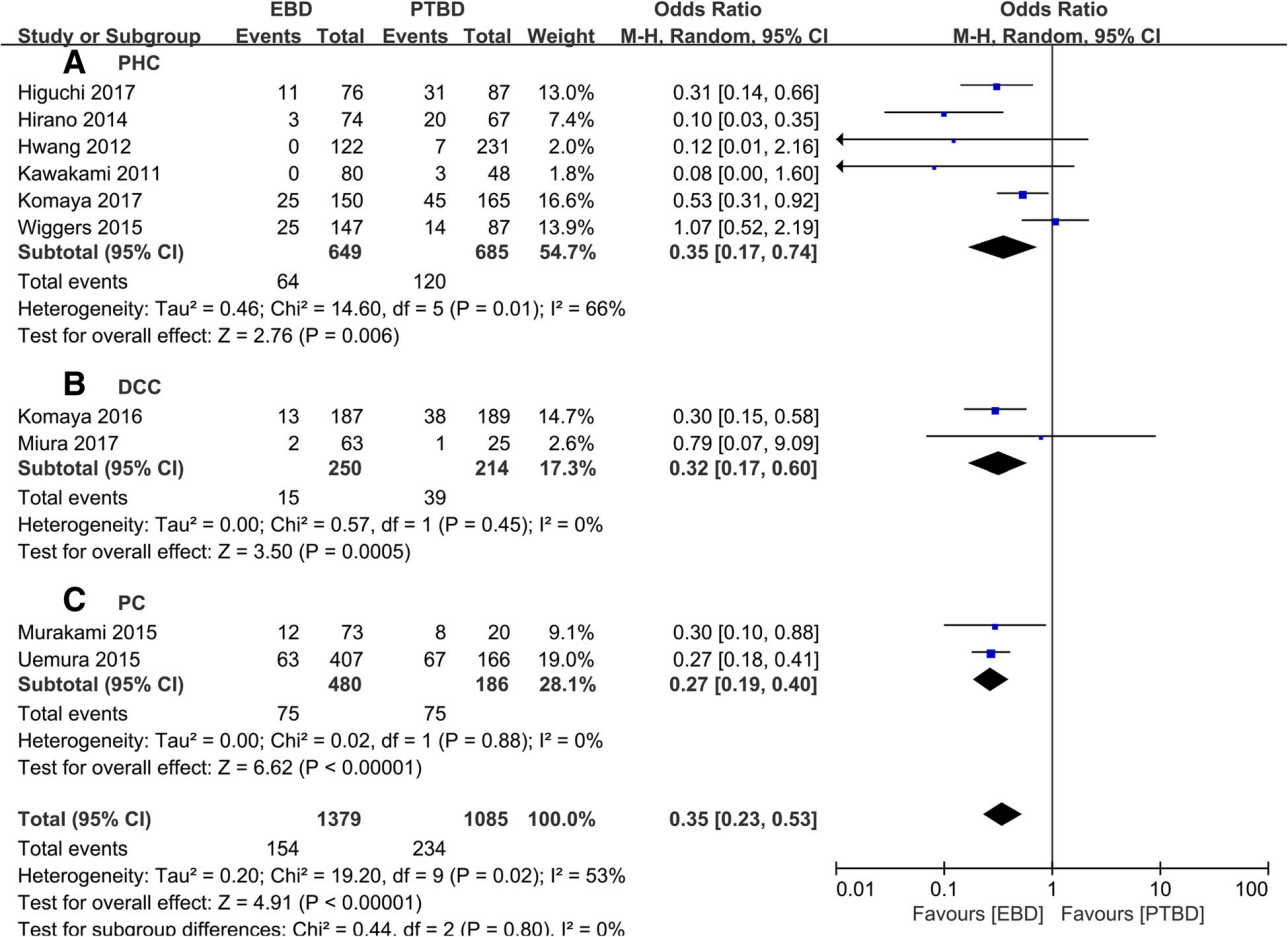
Subgroup analysis of seeding metastasis rates derived from (**a**) PHC, (**b**) DCC, and (**c**) PC

PHC, DCC, and PC were the mainly pathogenies for MBO, and subgroup results showed that, in the prevention of SM, EBD was superior to PTBD in PHC [10, 12, 15, 16, 18, 19] (7.8% vs. 17.1%, OR = 0.27, 95% CI 0.13~0.56, *P* < 0.001, Fig. 4(a)), DCC [13, 17] (6% vs. 18.2%, OR = 0.32, 95% CI 0.17~0.60, *P* < 0.001, Fig. 4(b)), and PC [11, 14] (15.6% vs. 40.3%, OR = 0.27, 95% CI 0.19~0.40, *P* < 0.001, Fig. 4(c)).

### Publication bias

**Fig. 5:**
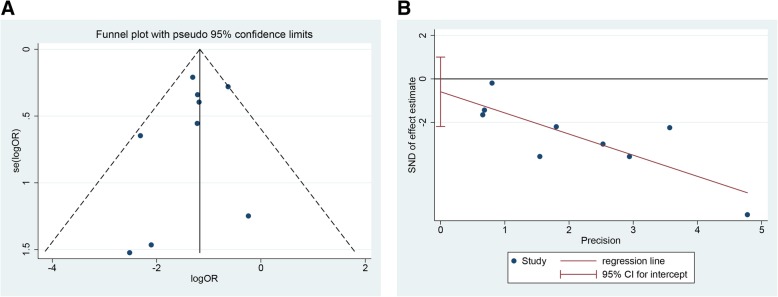
**a**, **b** Publication bias and sensitivity analysis

Funnel plot and Egger’s tests were used to detect the publication bias of our meta-analysis. A total of 10 studies [10–19] evaluating the seeding metastasis rate of MBO patients treated with EBD or PTBD exhibited a basically symmetrical funnel plot (Fig. 5a) and yielded an Egger’s test score of *P* = 0.409 (Fig. 5b).

## Discussion

This is the first systematic review focusing on the incidence of SM related to PBD for resectable MBO. A total of 10 studies with 2230 patients comparing the incidence of SM between PTBD and EBD were included in this study. Meta-analysis showed that EBD was associated with fewer SM than PTBD in the procedure of PBD for resectable MBO (10.5% vs. 22.0%, *P* < 0.00001). Hence, we concluded that EBD could be considered in patients with resectable MBO.

PHC, DCC, and PC are typically present with biliary obstruction, which often increases the risk of perioperative mortality and morbidity [21, 22]. Palliative biliary drainage has been repeatedly confirmed to be efficient in the improvement of prognosis for patients with unresectable MBO [23, 24], but it still remains controversial whether patients with resectable MBO would be benefited from PBD [2–4]. Furthermore, whether either PTBD or EBD is better is another puzzle [5–9], although both have been conducted prevalently worldwide. Short-term outcomes, such as pancreatitis, bile leakage, and clinical and technique success rates, are the common indicators to compare the efficacy and safety between PTBD and EBD. From this aspect, EBD has been confirmed superior to PTBD by several meta-analyses [25–27]. Long-term outcome of PBD was rarely taken into consideration of the strategy for patients with MBO, but the superiority of EBD in the overall survival was reconfirmed in our previous meta-analysis [28].

SM is rarely reported globally, but it was reported as high as 4.0~40.4% in Japan [10–17]. Reasons are as follows: (a) preoperative PTBD longer than 60 days was associated with an increased risk of the SM [29], (b) repeated attempts at PTBD [29, 30], and (c) multiple plastic stents were used rather than single one [29, 30]. In this study, eight included studies came from Japan [10–17], and the total incidence of SM (18.4%) was higher than that from Korea [19] (2.0%) and comparable with Netherland-USA [18] (16.7%). In the view of statistical data, SM was no longer an incident. Hence, the issue of SM deserved much more attention in clinical.

Theoretically, EBD is unlikely to cause SM. In this study, the incidence of SM is much lower in the EBD group than that in the PTBD group (10.5% vs. 22.0%, *P* < 0.001). Potential mechanism of increased PTBD-related SM lied in that as follows: (1) tumor cells derived from the PTBD fluid drainage were reported to be more than those from the EBD [19], which indicated that PTBD was much more likely to cause tumor diffusion. (2) PTBD was performed conventionally in the right liver, where the liver, peritoneum, diaphragm, pleura, and subcutaneous tissue were very close to each other anatomically [17], which offered an appropriate environment for SM. Hence, Takahashi et al. [29] recommended that PTBD should be performed in the left. (3) The general condition was usually much poorer in the patient intended to conduct PTBD, which meant a higher risk for SM.

The location of the MBO might be taken into consideration to decide an appropriate PBD. The recommended level of PTBD is higher than that of EBD for PHC according to the Chinese guideline (IIA vs. IIB) [31], but it remains controversial for DCC and PC in most of the guidelines [5, 6, 8]. In this study, the incidences of SM differed significantly among PHC, DCC, and PC (16.2% vs. 11.6%, and 22.5%), partly owing to varied aggressive characteristics of different cancers. However, subgroup analyses which were stratified by PHC, DCC, and PHC showed that the incidences of SM were lower in the EBD groups than those in the PTBD groups (7.8% vs. 11.7%, *P* < 0.001; 6.0% vs. 18.2%, *P* < 0.001; 15.6% vs. 40.3%, *P* < 0.001; respectively), which indicated that EBD was superior to PTBD in the prevention of SM regardless of the location of MBO.

However, we felt puzzled for the mechanism of SM caused by EBD. Technically, EBD was unlikely to cause peritoneal metastasis unless intestinal perforation occurred in the procedure of EBD, especially in the experienced centers. In this study, the overall incidence of tube-related SM in the EBD group is 2.0%. Theoretically, EBD was unlikely to cause peritoneal SM, because the whole procedure of EBD was conducted inside the biliary duct. In this study, we divided SM into tube-related SM and peritoneal SM, and subgroup analysis showed that both the rate of tube-related SM and peritoneal metastasis decreased significantly in the EBD group (2.0% vs. 6.7%, *P* < 0.001; 10.0% vs. 20.2%, *P* < 0.001; respectively). Hence, we concluded that EBD was superior to PTBD in the prophylaxis of SM, although the definition of SM remained controversial.

There were several limitations in this study. First, there were no RCTs included in this meta-analysis, which made the conclusion sound weaken because cohort data had selection bias. Second, studies included in this meta-analysis were nearly from Japan, which indicated obvious regional bias. Third, PTBD was available when EBD failed, but those patients belonged to which group remained inconsistent [15, 19]. Bias could be also due to the following: (a) the requirement for an alternative drainage procedure due to therapeutic or technical failures was likely higher in the EBD group compared to patients undergoing preoperative PTBD and (b) Bismuth type III and IV tumors as compared to type I and II tumors were potentially better decompressed percutaneously, especially in the presence of complex strictures. Fourth, the definition of SM varied from each other due to the lack of a golden standard, for example, intrahepatic metastasis belonged to PBD-related seeding metastasis in PC [14], but as for PHC and DCC, it tended to be rich in contradiction. Fifth, technical parameters of either PTBD or EBD, such as the procedure of PBD and the stent material, were different from each center, which indicated an inevitable heterogeneity and weaken the reliability. Sixth, the severity of obstructive jaundice, i.e., the level of preoperative serum bilirubin, was far from consistency, and caution should be taken when interpreting these results. The last but not the least, it was hard to avoid publication bias, because the journals tend to publish positive results.

In summary, we concluded that EBD was superior to PTBD for resectable MBO in the prophylaxis of SM, but there were currently not enough evidences. In the future, working out the definition of SM related to PBD is the urgent affair.

## Data Availability

All data generated or analyzed during this study are included in the published articles.
